# Values and DSM-5: looking at the debate on attenuated psychosis syndrome

**DOI:** 10.1186/s12910-016-0090-8

**Published:** 2016-01-20

**Authors:** Arthur Maciel Nunes Gonçalves, Clarissa de Rosalmeida Dantas, Claudio E. M. Banzato

**Affiliations:** Department of Psychiatry, Medical School, University of Campinas (UNICAMP), Rua Tessália Vieira de Camargo, 126 - Cidade Universitária, Campinas, 13083-887, SP Brazil

**Keywords:** Values, Ethics, Attenuated Psychosis Syndrome, DSM-5

## Abstract

**Background:**

Although values have increasingly received attention in psychiatric literature over the last three decades, their role has been only partially acknowledged in psychiatric classification endeavors. The review process of the fifth edition of the Diagnostic and Statistical Manual of Mental Disorders (DSM-5) received harsh criticism, and was even considered secretive by some authors. Also, it lacked an official discussion of values at play. In this perspective paper we briefly discuss the interplay of some values in the scientific and non-scientific debate around one of the most debated DSM-5 category proposals, the Attenuated Psychosis Syndrome (APS). Then, we point out some ethical consequences of a facts-plus-values perspective in psychiatric classification.

**Discussion:**

Different stakeholders participated in the APS-debate and for analytical purposes we divided them into four groups: (i) researchers in the field of high-risk mental states; (ii) the DSM-5 Psychotic Disorders Work Group; (iii) patient, carers and advocacy groups; and (iv) external stakeholders, not related to the previous groups, but which also publicly expressed their opinions about APS inclusion in DSM-5. We found that each group differently stressed the role of values we examined in the APS-debate. These values were ethical, but also epistemic, political, economic and ontological. The prominence given to some values, and the lack of discussion about others, generated divergent positions among stakeholders in the debate.

**Summary:**

As exemplified by the APS discussion, although medicine is primarily an ethical endeavor, values of different kinds that take part in it also shape to a large extent the profession. Thus, it may be strategic to openly discuss values at play in the elaboration of diagnostic tools and classificatory systems. This task, more than scientifically or politically significant, is ethically important.

## Background

Along with scientific data, values have increasingly received attention in psychiatric literature over the last three decades. Authors in philosophy of psychiatry have stressed the importance of values in psychiatric diagnosis, treatment, research and classification [1, 2]. Researchers in the field have developed a values-based framework for clinical practice and have also extensively argued in favor of a facts-plus-values perspective for healthcare [3, 4]. More than ever values have assumed the foreground of discussions in psychiatry and their diversity, if embraced, is believed to have great impact not only on psychiatry, but also on the future of medicine [3].

While facts and values are ontologically inseparable, their didactic distinction has been useful for understanding evaluative meanings in psychiatric discourse [3]. According to the eminent values scholar John Z. Sadler, values can be defined as guides to human action that are subject to praise or blame and that are present, implicit or explicitly, in all human activities [1]. Thus, they may pertain to different kinds, five of which at least proved to be useful in psychiatric classification values research [1, 5]: (a) aesthetic: related to notions of form and beauty; (b) epistemic: related to claims of knowledge; (c) ethical: related to goods, morality and virtue; (d) ontological: related to human nature and existence (and also the nature of mental disorders); and (e) pragmatic: related to the practical fulfillment of human actions, including administrative, logistic, political, economic and other practical values.

Values may also be described according to the way human action receives evaluative weight. They may be directly invested in (value-commitments), implied or assumed in an individual or group world-view (value-entailments) or weighted according to the effects of actions (value-consequences) [1]. From a linguistic perspective, values may be terms, which themselves present evaluative meaning, or semantics—sentences without value-terms, but with value-content [5]. Due to their multiplicity, values may conflict and it is especially when that happens that they become most visible [2]. According to the creator of the Values-Based Practice, K.W.M. (Bill) Fulford, to address values conflicts in a democratic scenario, stakeholders should openly discuss values at play and define which of those values should have precedence over others in a given context [6].

Although much has been recommended by values scholars, psychiatric classification endeavors still seem to only partially acknowledge the role of values. For instance, some critics of the fifth edition of the Diagnostic and Statistical Manual of Mental Disorders (DSM-5) claimed its review process was conducted in a “secretive” way, lacking “adequate public record of the rationale for changes” [7]. Although the American Psychiatric Association (APA) published drafts of DSM-5 criteria for online public feedback and declared interest in closer collaboration with external stakeholders, including patient advocacy groups [8], it did not publish the online comments themselves, nor disclosed how each DSM-5 work group considered and incorporated the feedback [9]. Also, no official discussion about values at play was reported.

In this perspective paper, we briefly discuss the interplay of some values in the scientific and non-scientific debate around one of the most debated DSM-5 category proposals, the Attenuated Psychosis Syndrome (APS). The debate surrounding this proposal was heated, full of conflicting values, and APS ended up not being coded as an official diagnosis in DSM-5 [10]. We begin by discussing APS history and the groups of stakeholders involved in its debate. Then, we present some values that were operative, even when unacknowledged, within this debate by sorting them out according to Sadler’s values typology. After, we discuss their interplay, highlighting the contexts in which they appeared and by whom they were put in action. We conclude by pointing out some ethical consequences of a facts-plus-values perspective in psychiatric classification.

## Discussion

### APS early history

Considered one of the most severe psychiatric syndromes, psychosis still evokes a “corrosive pessimism” among psychiatrists [11] and its treatment still is frequently seen as a palliative aid to patients [12]. The long history of therapeutic limitation regarding this diagnosis provided the impetus to early interventions efforts in the field [13]. However, according to some researchers, decreasing the duration of untreated psychosis has provided only modest improvements in outcome for individuals with schizophrenia, leading to interest in possibilities for intervention before psychosis onset [13, 14]. In mid 1990s this interest led to the development of the high-risk mental state concept, which pioneered in Australia, but was quickly adopted and further developed in many specialized research centers around the world, spanning more than 11 countries [15].

Early in the preparation of DSM-5, the Psychotic Disorders Work Group proposed the creation of a new diagnosis to serve as a placeholder for the high-risk mental state concept [16]. The proposal was firstly termed Psychosis Risk Syndrome (PRS) and was criticized as a premature and confusing category. After all, studies showed that individuals at risk already needed treatment for their current psychiatric symptoms and not only the potential preventive effect of early intervention on a “risk syndrome” [17]. Thus, the new category proposal was reconsidered as a mental disorder per se. It was renamed APS, according to the term assigned to one subgroup of patients identified by the high-risk mental state research criteria [18]. Although other sets of high-risk criteria existed, most at-risk patients identified in specialized research centers presented APS as their main medical complaint. The syndrome differed from full-blown psychosis due to the sub-threshold (attenuated) intensity or frequency of presented symptoms [16].

After the name change, the debate about including or not APS in DSM-5 proceeded with diverse arguments. Even after DSM-5 task-force rejected APS from the manual main section, debaters still continued discussing whether the proposal was ready for DSM-5.1 or 5.2 [15, 16].

### Stakeholders

Both in scientific literature and in non-scientific publications different stakeholders participated in the APS-debate. For analytical purposes and in order to explore the wide range of values at play in this debate we selected some stakeholders that presented influential and diverse opinions, dividing them into four groups. Even though a prevalent view could be identified within each group, we do not assume that there was no dissonance within them. We also do not assume that our selection of stakeholders is exhaustive of the diversity of participants and opinions in the APS-debate. The first group was composed by (i) high-risk mental state researchers, which have had differences in opinion about to what extent their findings were represented by the APS proposal and, consequently, about the merits of including APS as a new diagnosis in DSM-5 [19]. As a point of consensus, however, all of them considered APS a useful concept and supported its inclusion at least as a “condition for further study” in Section III of DSM-5 [20]. Another group was represented by the (ii) Psychotic Disorders Work Group itself, which proposed and evaluated the APS construct along the DSM-5 review process. Composed by 12 members, the Work Group nominees were approved in 2008 by the APA Board of Trustees [21]. All members were active researchers on schizophrenia, nine from USA and three from Germany, Netherlands and UK [13]. In addition to reviewing all available data about high-risk mental states, the Work Group consulted a range of experts and considered public and expert comments on APS proposal. After DSM-5 field trials, group members determined that more work was necessary before APS could be considered for inclusion in the main body of the manual. Thus, they recommended its inclusion with specific criteria and description in Section III, a part of the manual that harbors proposed conditions for further research [13].

A third group of stakeholders was composed by (iii) patients, carers and advocacy organizations. Although individual patients and carers had the opportunity to post online comments on DSM-5 drafts through the website dedicated to the manual [22], direct conversation with APA Board of Trustees was performed mainly by patient advocacy organizations [9]. One of the institutions involved in the APS-debate was the National Alliance on Mental Illness (NAMI), a well-established North-American non-profit organization founded in 1979 [23]. With over 200,000 members and expenditures of over US$8.1 million on program and membership support, educational services and patient advocacy [24], NAMI maintained close connections to APA [9]. During the debate on APS, this organization supported the inclusion of the proposal as an official diagnosis in DSM-5 main section [25].

Other authors outside the field of high-risk mental states and also not involved with APA, DSM-5 task-force, the Work Group or patient advocacy groups, publicly expressed their opinions about APS inclusion. Among this group of (iv) external stakeholders was Allen Frances, chairman of DSM-IV task-force, who explicitly opposed APS, considering it the worst DSM-5 proposal [26]. Frances strongly advocated its inclusion in the manual appendix, especially after realizing APS had real chances to become an official DSM-5 diagnosis [27].

### Values guiding arguments in favor of APS inclusion

#### Relieving suffering and preventing psychosis

According to APS advocates, including it as an official diagnosis could expand access to appropriate care for individuals around the globe suffering with this condition. Associated symptoms could be treated, including anxiety, depressive symptoms, social withdrawal, and academic impairment, even for those patients who would never become psychotic [28]. Also, clinical recognition of APS could hypothetically reduce the rates of misdiagnoses, improving the process of differential diagnosis, promoting better case management and providing proper reimbursement [18].

Among the stakeholders who acknowledged APS potential benefits to patients were researchers in the field of high-risk mental states, which considered that the new proposal could be a “clarion call to the field to focus attention on these patients and families *in need*” [20]^1^. NAMI also overtly supported APS inclusion, as noted by the organization’s list of potential and intended benefits with this diagnosis: “possibly less severe symptoms, more rapid recovery, prevention of the most *devastating* consequences of lack of treatment such as homelessness, criminal justice involvement, and suicides” [25]. In NAMI’s opinion, and according to one of its institutional aims, making APS an official diagnosis could also be useful on illness education and support for individuals at risk and family members.

Even critics of APS such as Allen Frances implicitly recognized that the Work Group “has always been well intentioned” on the proposal [27], as the “*debilitating*” nature of psychosis with its “*profound lifelong impairments*” has never been put in question [13]. The Work Group hoped that by targeting APS secondary prevention of full psychosis would take place, possibly offering “substantial *life course benefits*” to patients [13]. William Carpenter, the chairman of the Work Group and an eminent researcher on schizophrenia, declared his personal view that:

[t]here is much that clinicians *can and should do* for care-seeking individuals with *distress and dysfunction* who manifest early psychotic-like psychopathology. A new DSM-5 diagnosis can focus attention on this syndrome and stimulate the creative acquisition of new knowledge that may be *life altering for afflicted persons* [29].

Efforts to improve patients’ quality of life by trying to prevent later psychosis and treating current symptoms are intimately related to medicine’s core value of aiding the ill [1]. After all, patient’s vulnerability, the intrinsic undesirability of diseases and disorders, and the professional commitment to aid the ill are the elements that form the ethical, value-laden core of medicine and related health professions [1, 30]. Carpenter’s quotation overtly presented the value of aiding the ill and summoned the position of researchers in the field, the Work Group, and NAMI, who considered APS inclusion as an ethical value-commitment to aid patients in suffer.

As discussed elsewhere [1], the explicit commitment to aid the ill is not directly found in DSMs, in which value-commitments are primarily geared towards aiding clinicians in their practice (thus, indirectly aiding patients). This position (i.e., “clinical usefulness” representing “aiding the ill”) was still valid in DSM-5, as stated on its preface: “this edition of DSM was designed first and foremost to be a useful guide to clinical practice” [10]. APS text and criteria in Section III also reinforced this position by the apparently more descriptive than evaluative role of value-terms like “distressing”, “disabling”, and “impaired function” [10].

The degree of importance given by each actor to this and other values in the debate and how they compared to values in DSM-5 are illustrated on Table 1.

**Table 1:** Importance given in discourse to some values according to group of stakeholders in the APS-debate and to the DSM-5

		Researchers in high-risk mental states	Psychotic Disorders Work Group	Patient advocacy groups (NAMI)	External stakeholders (Allen Frances)	DSM-5
Value-commitments						
Aiding the ill by relieving suffering and preventing psychosis	[ethical]	+++	+++	+++	+	+
Scientific development by research expansion and knowledge acquisition	[epistemic/pragmatic]	++	++	-	-	+
Value-consequences						
Stigmatization and inappropriate prescription of antipsychotic medication	[ethical]	++(+)	++	+++	+++	-
Value-entailments						
Mental health commercialism and the influence of political and economic forces	[ontological/pragmatic]	(++)	-	++	+++	-
Neurobiological reductionism and reification	[ontological]	(++)	-	-	-	-

- : absent; + : implicit; ++ : explicit; +++ : emphasized; ( ) : importance given by part of the group

#### Research expansion and knowledge acquisition

As already demonstrated by Carpenter’s quotation, some advocates of APS inclusion in DSM-5 also argued that this move could “stimulate the creative acquisition of new knowledge” in the field of high-risk mental states [29]. To them, although research on the APS concept was already steadily growing in specialized centers, making this construct an official category listed in a globally used psychiatric classification could give the proposal more visibility, fostering discoveries in the field and providing more research funding [18, 31]. Psychiatrists in specialized centers argued that research into APS group of patients could have the potential to “*increase our understanding* of psychotic-like symptoms and their trajectories and the emerging phase of psychotic disorders” [20]. In their opinion APS inclusion would also bring psychiatry “in line with other fields of medicine that identify risk factors for the purposes of instituting preventative interventions” [28].

The Work Group also presented interest in research expansion in the field of high-risk mental states, an apparently natural consequence of the fact that its members were also active researchers on psychotic disorders and had the task to develop a manual with multiple purposes, including that to be “a reference for researchers” [10]. The Work Group recognized that “early detection and intervention is a high value throughout medicine” and speculated that “psychiatry will move in this direction with a number of disorders in the future” [13]. After DSM-5 field trials failed to show APS diagnostic reliability in clinical settings, Work Group members still “concluded that there were strong reasons to continue to evaluate this clinical entity” and then recommended APS assignment to Section III for further study [13]. The referred section harbored proposals “on which future research is encouraged” with hope that “such research *will allow the field to better understand* these conditions” [10].

From an evaluative perspective, research expansion and knowledge acquisition represented another value-commitment related to APS inclusion, that of scientific development of high-risk mental states field and of psychiatry itself. This value-commitment had both an epistemic and a political nature. Epistemic in the sense that it was related to a specifically desired way to structure knowledge (i.e., scientifically). Political in the sense that by aligning psychiatry with other medical specialties that perform preventive interventions it would improve the medical status of psychiatric field, a position questioned from time to time in the history of the specialty [32] by those who consider it scientifically underdeveloped [3].

Both researchers and Work Group members were committed to the value of scientific development. Due to their standpoints, the ethical and the epistemic value-commitments were naturally intertwined in their discourses. Specifically in the case of the DSM-5 text, the epistemic value-commitment of scientific development was intertwined with that of the already mentioned “clinical usefulness”. Both NAMI and Frances did not value APS as a commitment to scientific development.

### Values guiding arguments against APS inclusion

#### Stigmatization and inappropriate prescription of antipsychotic medication

On the other side of the debate, APS opponents argued that this diagnosis could undesirably stigmatize and generate unnecessary treatment to youngsters whose majority would never transition to psychosis [16]. To these critics, making APS an official diagnostic category could also promote inappropriate allocation of the already scarce resources destined to mental health and bring incalculable damages to society [9, 18, 27]. Potential inadequate prescription of antipsychotic medication, with their harmful effects of weight gain and increased cardiovascular risk, could cause a profound impact on the life of identified population. Moreover, stigmatizing effects could be unpredictable on the individually and socially perceived sense of autonomy and responsibility of diagnosed patients [27, 33].

Researchers in the field recognized the possibility of stigma, discrimination and inappropriate prescription of antipsychotic medication as one element of the risk-benefit analysis of APS inclusion in DSM-5 [18]. In a collective paper reporting points of consensus of their analysis, they agreed that “on current evidence, antipsychotic medication is no more effective than other *more benign* treatments and so is typically not recommended” [20]. Regarding stigma, in their opinion “the effects of creating a new diagnosis, on patients, their families, and the wider health system, needs to be better understood” [20].

The Work Group also acknowledged these potential negative effects of APS inclusion. In its official publication, however, the group even presented a counter-argument found in the literature to these concerns: “any stigma is principally related to behaviors associated with a diagnosis of APS rather than the diagnosis itself” [13]. Furthermore, according to the counter-argument presented, “a new APS category will educate clinicians about the relative lack of utility of antipsychotic medications in this population and may actually reduce inappropriate antipsychotic use among youth” [13]. However, Carpenter’s defense of APS inclusion as an official category partially hinged on the prospects of the development of safer medicines. Carpenter made it clear that if that happened he “would have less concern about whether or not some harm is done to people who are included in the syndrome” [34]. Thus, the very availability of medicines could modulate the threshold for including a new category in DSM-5.

Different from previous stakeholders, NAMI not only acknowledged, but also emphasized potential negative effects of making APS an official category. Weighing risks and benefits, the organization supported APS inclusion, but suggested it should be accompanied with special care to educate clinicians that psychosocial interventions and support, including family education, were first line strategies for responding to diagnosed individuals. To NAMI “threshold for prescribing antipsychotic medications should be very high” [25]. Also, according to the organization “clinicians and allied mental health professionals must be aware and particularly sensitive to the *potential negative implications* of affixing a diagnosis” [25]. NAMI defended that these safeguards should be implemented to protect the privacy of individuals and their families, avoiding “over use of this diagnosis” [25].

In the external stakeholders group, Allen Frances also stressed the unpredictable effects of APS inclusion as a DSM-5 category. For him, “the treatment most likely to be used would be antipsychotic medications” which “have no proven efficacy in preventing psychosis, but most definitely have terrible side effects” [27]. Frances stated as a special point of concern that antipsychotic medication would be “overprescribed to those least able to resist-the young and those who are most financially disadvantaged” [27]. Regarding stigma he considered that “having a label that suggests one is at risk to soon develop a psychosis would cause the mislabeled person much unnecessary *worry*, unnecessarily *reduced ambitions*, and create great *risk of discrimination*” [27].

APS opponents warned that including this category in DSM-5 could perform exactly the opposite effect to that of aiding the ill. Thus, both stigmatization and inappropriate prescription of antipsychotic medication were weighted in the debate as negative ethical value-consequences. Although they were discussed together by most groups of stakeholders, APS text in DSM-5 did not mention these possible negative value-consequences [22, 35].

### Contextual values

#### Influence of social forces

According to Yung and Nelson, expert researchers of the Australian group on prevention and early intervention for psychosis, another point of concern regarding APS inclusion was related to the phenomenon of “diagnostic creep,” i.e., the “gradually shifting of the threshold for a diagnosis in response to clinical practice, political lobbying, and other social forces” [18]. One possible example of “diagnostic creep” would be of a psychiatrist making an APS diagnosis to a patient with mild psychotic symptoms, but technically not meeting enough criteria, motivated by insurance reimbursement and other benefits derived from treatment access through a formal diagnosis [18, 33]. The authors and colleagues also questioned if the “rigidity of the US health insurance system, which deems a DSM diagnosis necessary but not sufficient for coverage, [should] determine what conditions are seen as psychiatric disorders” [33]. To these researchers, social forces could potentially alter APS diagnostic threshold in clinical practice independently of clinical reasons.

NAMI also acknowledged the role of the referred social forces in the APS-debate. After suggesting safeguards for APS inclusion in DSM-5, the organization asked: “will these [recommended] practices be followed in the current health care environment where insurance companies frequently limit reimbursement for care and treatment and may favor treatment with medication over psychosocial interventions?” [25].

Allen Frances heavily stressed the role of social forces in the APS-debate especially in the figure of pharmaceutical companies. He acknowledged that it was not the intention of the Work Group that APS be initially treated with antipsychotic medication, but in his opinion “experience teaches clearly that drug companies will successfully exploit any new diagnosis in their unremitting efforts to boost already swollen sales” [26]. For Frances, it would not even be safe to rely on professional education to reduce the risks of antipsychotic medication overprescription, as “most of the physician education come from the very drug companies” [27]. However, most researchers in the field, the Work Group, and APS text in DSM-5 did not discuss these external influences on diagnosis.

In the APS-debate the social forces influencing diagnostic threshold mainly assumed the form of political and economic interests. These forces closely related to the concept discussed elsewhere [31] of a mental health-medical-industrial complex, in which different interests act to expand already established political and economic power structures [1, 31]. These forces operate based on a perspective that conceives mental health as a commercial field, subject to market laws and as a means for profiting [31]. According to this perspective patients are consumers and medical costs are financial losses to insurers. This mental health commercialism represents one form, among many, of understanding human beings, mental illness and its treatment, and is, thus, an ontological value-entailment, mostly undiscussed in the APS-debate.

#### Neurobiological reductionism and reification

Although many expert researchers in the field initially argued in favor of APS as a potentially beneficial category, some of them had withdrawn their support especially due to the lack of consensus about how, in practice, psychiatrists would treat identified patients [26]. In addition to the influence of economic and political forces, the lack of agreement about which model of “aiding the ill” would be adopted to treat APS patients also increased the perception of risks related to making this proposal an official diagnosis. Regarding the issue of models of care, Patrick McGorry, pioneer in high-risk mental states research and also member of the Australian group, pointed out the need to break “the nexus in the U.S. that drug treatment is the main or only form of intervention for patients — a nexus reinforced by the hard neurobiological reductionism that took over American psychiatry from the 1980s” [36].

In the same vein, Yung and colleagues highlighted the concern of APS “reification” [33] if this proposal ended up being coded an official DSM-5 category. When a diagnostic concept is officially listed in a classification manual and presented with an operational definition, there is a trend to gradually reify^2^ this construct [37, 38]. Among the risks of psychiatric categories reification is the limiting of scientific advances, either by preferential funding of researches that use these categories, either by transforming the diagnoses in epistemic blinders that hinder theoretical formulation (or even imagination) of alternative models of mental illness [31, 37]. Another risk associated to reification relates to “framing” of subjective experience according to reified disorder categories. As discussed elsewhere, “reifying aspects of our mental life modifies the world in which we live in and may change who we are and what we do” [39].

In APS context, hard neurobiological reductionism and reification were also value-entailments regarding the ontology of mental disorders and their diagnosis. They also represented chosen ways of conceiving what are human beings and mental illnesses, what is their nature (e.g., biological, psychological, social), where and how illnesses manifest, what are their causes, and, consequently, how they should be treated.

If the unavailability of safer medicines modulated the threshold for making or not APS an official diagnosis, the lack of discussion on the ontological perspective underlying DSM-5 and psychiatry in general, and the influence of economic and political forces, both cast doubt into which would be the threshold for choosing medicines over other therapies. Although these thresholds are theoretically attributed to the influence of epistemic values, such as scientific rigor and evidence-based medicine, studies have shown a trend in psychiatry towards use of medications even in disagreement with clinical guidelines and current scientific evidence [19]. This observation illustrates two important points: first, how different kinds of values (e.g., pragmatic and ontological) may work together to influence a decision; and, second, how there is no guarantee that values expected to play the highest priority roles in a given situation (e.g., epistemic) will actually do it.

Most researchers in the field, NAMI, Allen Frances and the Work Group did not mention the value-entailments of neurobiological reductionism and reification in the APS-debate.

### APS out of DSM-5 main text

Despite of a long and intense debate full of conflicting values, APA official justification for rejecting APS from DSM-5 main text was the failure of field trials to recruit a large enough sample of patients to measure APS diagnostic reliability [40]. APA published results from field studies in January 2013 on two papers written by Darrel Regier, vice-president of DSM-5 task-force, and other members [40, 41]. In the second paper authors declared that, although the sampling method used in DSM-5 field trials yielded greater statistical power than in previous DSM studies, it did not obtain adequate sample sizes for some conditions. The “difficulty in recruiting patients with relatively low prevalence mental disorders [including APS] was not fully appreciated by either the [research] sites or the investigators” [40]. In the first article, authors stated that “the results of the DSM-5 Field Trials were intended to help to inform the DSM-5 decision-making process (along with many other factors unrelated to field trials)” [41]. Nevertheless, APA did not present other reasons for APS rejection beyond the unknown diagnostic reliability, nor discussed, to our knowledge, the other factors involved in this decision.

As demonstrated through our brief exploration of values in the APS-debate, the lack of an official discussion about values at play on DSM-5 elaboration process was mirrored by the manual final text [7], still written with a “value-free” and “objectivist” approach [3, 42].

### Ethical consequences of a facts-plus-values perspective in psychiatric classification

Medicine, including psychiatry, is a moral enterprise, and has been so regarded since Hippocratic times [30]. However, as we have seen through the APS-debate, ethical values represent only one kind of values at stake in medical practice. We argue that although ethical values are by far the most acknowledged and praised ones, epistemic, aesthetic, pragmatic and ontological values are no less important in guiding professional actions, including clinical activities, medical research, and psychiatric classification [5]. If this argument is true, exploring different kinds of values at play is essential to correctly identify top-priority values and, consequently, to acknowledge and address potential value-disputes in a given situation or endeavor.

As we have seen so far, the lack of a fully open, transparent, and fact-plus-values informed review process may have opened DSM-5 to harsh criticism. Regarding APS rejection from DSM-5 main section, the disclosure of only one reason by APA brought many questions to the fore. If unknown diagnostic reliability solely accounted for rejecting APS, was it more important than the previously mentioned potential unintended consequences of this new category? Did APA also fear those consequences? If so, why not officially disclosing this concern?

In the APS-debate even patient involvement, which represented a new historical step in DSM elaboration, raised important questions, such as to what extent political and economic values may influence the implementation of scientific treatment guidelines for psychiatric conditions in clinical practice. Also, a most important question arose, that of what are stakeholders’ assumptions about the nature of mental illnesses, about what represents a good treatment, and, ultimately, about what constitutes a “good life” worth living by patients [1, 30].

## Conclusion

Multiple values permeated the debate about making or not APS an official DSM-5 diagnosis, including ethical, but also epistemic, political, economic and ontological ones. The prominence given to some values, and the lack of discussion about others, generated divergent positions among researchers, clinicians, patient advocacy groups and other stakeholders. As has already been argued elsewhere [1], many critiques to the DSMs and their review processes precisely arise from the lack of open discussion about values that remain implicit. As demonstrated by the APS-debate, although medicine is primarily an ethical endeavor, values of different kinds that take part in it also shape the profession. Thus, it may be strategic to openly discuss values at play in the elaboration of diagnostic tools and classificatory systems. This task, more than scientifically or politically significant, is ethically important.

## Abbreviations

APA: American Psychiatric Association
APS: Attenuated Psychosis Syndrome
DSM-5: Diagnostic and Statistical Manual of Mental Disorders — fifth edition
NAMI: National Alliance on Mental Illness
PRS: Psychosis Risk Syndrome

## Footnotes

1 We added emphasis to quotations in order to highlight evaluative content. All emphases are ours.

2 Reification is understood in this context as a “misplaced concreteness” [39], making psychiatric diagnoses to be thought of, and used as, concrete objects, entities having independent existence on their own, instead of abstract and hypothetical formulations about reality [37, 39].
